# Atomic models of microtubule doublets and dyneins in cilia revealed by cryo-electron microscopy

**DOI:** 10.1186/2046-2530-4-S1-O6

**Published:** 2015-07-13

**Authors:** T Ishikawa, A Maheshwari, J Obbineni

**Affiliations:** 1 Laboratory of Biomolecular Research, Paul Scherrer Institute, Villigen, Switzerland

## 

Microtubule doublets (MTD) in cilia consist of the A-tubule (13 protofilaments) and the B-tubule (likely 10 protofilaments). While 3D molecular arrangement of ciliary proteins is our interest, our knowledge about how dyneins, radial spokes and other proteins bind to MTD was limited, due to the limitation of resolution. We have been investigating the conformation and localization of inner and outer dynein isoforms (Bui et al. (2012) JCB 198, 913) and their conformational change induced by ATP (Movassagh et al. (2010) NSMB 17, 761), radial spokes (Pigino et al. (2011) JCB 195, 673) by cryo-electron tomography (resolution 30A). We newly employed single particle cryo-EM technique and improved resolution to 20A. This enabled us to locate tubulin isoforms and describe interaction and binding of dyneins and other proteins precisely. The arrangement of tubulin dimers in MTD demonstrates difference from the reconstituted microtubule, indicating specific building mechanism of MTDs. Microtubule binding domains (MTBD) of outer arm dyneins bind to the three adjacent protofilaments, between alpha and beta-tubulins within one dimer. The orientation of the stalk changes ~5 degrees during the power stroke caused by rearrangement of the linker domain of dynein with respect to the AAA-ring as we reported (Ueno et al. (2014) Cytoskeleton 71, 412). MTBD of inner arm dyneins bind to one protofilament of the B-tubule, with the exception of dimeric dynein f. The interface of N-terminal tails of inner dyneins and the A-tubule spans over two protofilaments. We will extend our discussion of how this architecture is maintained during the bending motion.

